# 62 Cognitive Behavioral Therapy Improves Symptoms of Depression in Burn Survivors

**DOI:** 10.1093/jbcr/irae036.054

**Published:** 2024-04-17

**Authors:** Mikki J Rothbauer, Zuzanna Pasek, Sandi S Wewerka, Kirsten A Dalrymple, Nell Adams

**Affiliations:** HealthPartners, St. Paul, Minnesota; HealthPartners, St. Paul, Minnesota; HealthPartners, St. Paul, Minnesota; HealthPartners, St. Paul, Minnesota; HealthPartners, St. Paul, Minnesota

## Abstract

**Introduction:**

Burn injury patients can suffer severe trauma as they cope with the events that led to their injuries and their new physical abilities and appearance. Mental health is a component of care that is often overlooked for these patients. The purpose of this prospective study was to evaluate whether burn psychotherapy is effective in reducing depression in burn survivors.

**Methods:**

Our Burn Center has a full time licensed clinical psychotherapist who uses Cognitive Behavioral Therapy (CBT) to treat patients. Burn clinic nurses administered the Patient Health Questionnaire-9 (PHQ-9) depression screener to n=1842 patients between 2016-2019. Patients were referred to psychotherapy if their total PHQ-9 score met the definition for moderate depression (total score ≥ 10) or they endorsed the question about suicidal thoughts. Patients could also participate through self-referral.

Our final sample included n=146 patients (47% female, mean age=41.8) who attended at least one psychotherapy appointment and contributed at least one post-appointment PHQ-9 score. We computed a difference score for each patient by subtracting their baseline PHQ-9 score from their mean post-psychotherapy score. We used a one sample t-test to compare the mean difference score for the group to zero (zero = no change). We also examined how many participants showed a change in PHQ-9 category from pre- to post-psychotherapy.

**Results:**

The mean baseline score was 11.49 (SD = 6.92) vs. mean post-psychotherapy score 8.54 (SD = 6.45). The mean group difference score was significantly different from zero (mean = -2.95, SD=5.72), t(145)=-6.23, p< 0.001, [95% CI = -3.88 to -2.01]. The negative mean difference indicates a decrease in depressive symptoms. When examining changes in depression category, 50.7% of patients moved to a less severe category of depression after psychotherapy, 13.7% of patients moved to a more severe category, and 35.6% did not change.

**Conclusions:**

These results support psychotherapy as a measurable benefit to some, but not all individuals undergoing specialized burn care. Burn centers should consider having a full-time burn- and trauma-trained psychotherapist on staff to complete assessments on admission. Continuity of mental health care in burn centers has the potential to positively influence patient outcomes.

**Applicability of Research to Practice:**

Cognitive behavioral therapy is used in many different populations. Our findings support the practice of specialized psychotherapy intervention in the burn population as effective in decreasing symptoms of depression in some patients.

